# Dicer1 downregulation by multiple myeloma cells promotes the senescence and tumor-supporting capacity and decreases the differentiation potential of mesenchymal stem cells

**DOI:** 10.1038/s41419-018-0545-6

**Published:** 2018-05-03

**Authors:** Juan Guo, Youshan Zhao, Chengming Fei, Sida Zhao, Qingqing Zheng, Jiying Su, Dong Wu, Xiao Li, Chunkang Chang

**Affiliations:** Department of Hematology, Shanghai Jiao Tong University Affiliated Sixth People’s Hospital, 200233 Shanghai, China

## Abstract

Bone marrow mesenchymal stem cells (BMMSCs) facilitate the growth of multiple myeloma (MM) cells, but the underlying mechanisms remain unclear. This study demonstrates that the senescence of MM-MSCs significantly increased, as evidenced by a decrease in proliferation and increase in the number of cells positive for senescence-associated β-galactosidase activity. Senescent MM-MSCs displayed decreased differentiation potential and increased tumor-supporting capacity. Dicer1 knockdown in the MSCs of healthy controls promoted cellular senescence and tumor-supporting capacity, while decreasing the differentiation capacity. Dicer1 overexpression in MM-MSCs reversed the effects on differentiation and reduced cellular senescence. In addition, decreased expression of the microRNA-17 family was identified as a favorable element responsible for increasing senescence, with the expression of *p21* increased in Dicer1 knockdown cells. Furthermore, we observed decreased expression of miR-93 and miR-20a in MM-MSCs, while upregulation of miR-93/miR-20a decreased cellular senescence, as evidenced by the increased *p21* expression. Importantly, we found that myeloma cells could induce the senescence of MSCs from healthy controls, as observed from the decreased expression of Dicer1 and miR-93/miR-20a and increased expression of *p21*. Overall, MM cells downregulate Dicer1 in MSCs, which leads to senescence; in turn, senescent MSCs promote MM cell growth, which most likely contributes to disease progression.

## Introduction

Multiple myeloma (MM) is a malignancy characterized by plasma cell proliferation, initially in the bone marrow microenvironment^1^. This tissue is a complicated network of extracellular matrix and various cells^2^. Undifferentiated and pluripotent bone marrow mesenchymal stem cells (BMMSCs) are the principle members of the bone marrow microenvironment. BMMSCs promote MM cell proliferation via different factors, leading to increased tumor supporting capacity and the development of drug resistance. Evidence has shown that MSCs isolated from MM (MM-MSCs) exhibit different behavior from that exhibited by the MSCs from healthy controls (HC-MSCs), including differential gene expression, impaired cytokine release, and decreased osteogenic differentiation potential^3–6^. In MM, the interaction between BMMSCs and MM cells plays a pivotal role in MM development. MM cells attach to BMMSCs and release cytokines that promote MM cell viability, migration, and invasive ability, ultimately enhancing MM progression^7^. This interaction usually leads to complicated changes, including dysregulation of mRNA and miRNA expression and activation or deactivation of signaling pathways, but the underlying mechanisms remain unclear. For example, the development of a senescence-like state in BMMSCs, and thereby a modulated secretory profile, has been reported to worsen osteogenic differentiation potential^8–11^. Senescence is a cellular state corresponding with a decrease in proliferation ability and variation in the release of pro-inflammatory cytokines and growth factors^12^. Senescent BMMSCs exhibit an increased senescence-associated β-galactosidase activity (SA-β-gal) and irregular cell morphology^13, 14^. Generally, cell cycle of the senescent cells is often arrested at the G_0_/G_1_ phase, in combination with the upregulation of different cell cycle inhibitors such as p21^15–17^. Increasing body of report suggests that microRNAs (miRNAs) take part in the regulation of MSC senescence^18, 19^. Furthermore, they play an important part in the self-renewal and differentiation of MSCs. As previously indicated, MM-MSCs show a different miRNA profile to that of HC-MSCs, but the roles of these deregulated miRNAs in MM-MSCs are not clearly known^20, 21^. The loss of Dicer1, an RNAse III endonuclease essential for miRNA biogenesis, and the ablation of mature miRNA result in the inhibition of cell proliferation and the induction of a premature senescence phenotype^22–24^. Our previous study showed that Dicer1 expression is reduced in MSCs from myelodysplastic syndrome patients (MDS) and that this down-regulation promotes cellular senescence and decreases the stem cell-supporting capacity of these cells^17^. However, the effect of the Dicer1 gene on the pathogenesis of MM has not been studied.

In this study, we studied the senescent features of BMMSCs derived from MM patients and explored the biological function of Dicer1 in the senescence of MM-MSCs. Finally, we analyzed the decreased differentiation potential and the enhanced tumor-supporting capacity of MM-MSCs.

## Results

### Senescent features of BMMSCs from patients with MM

**Fig. 1:**
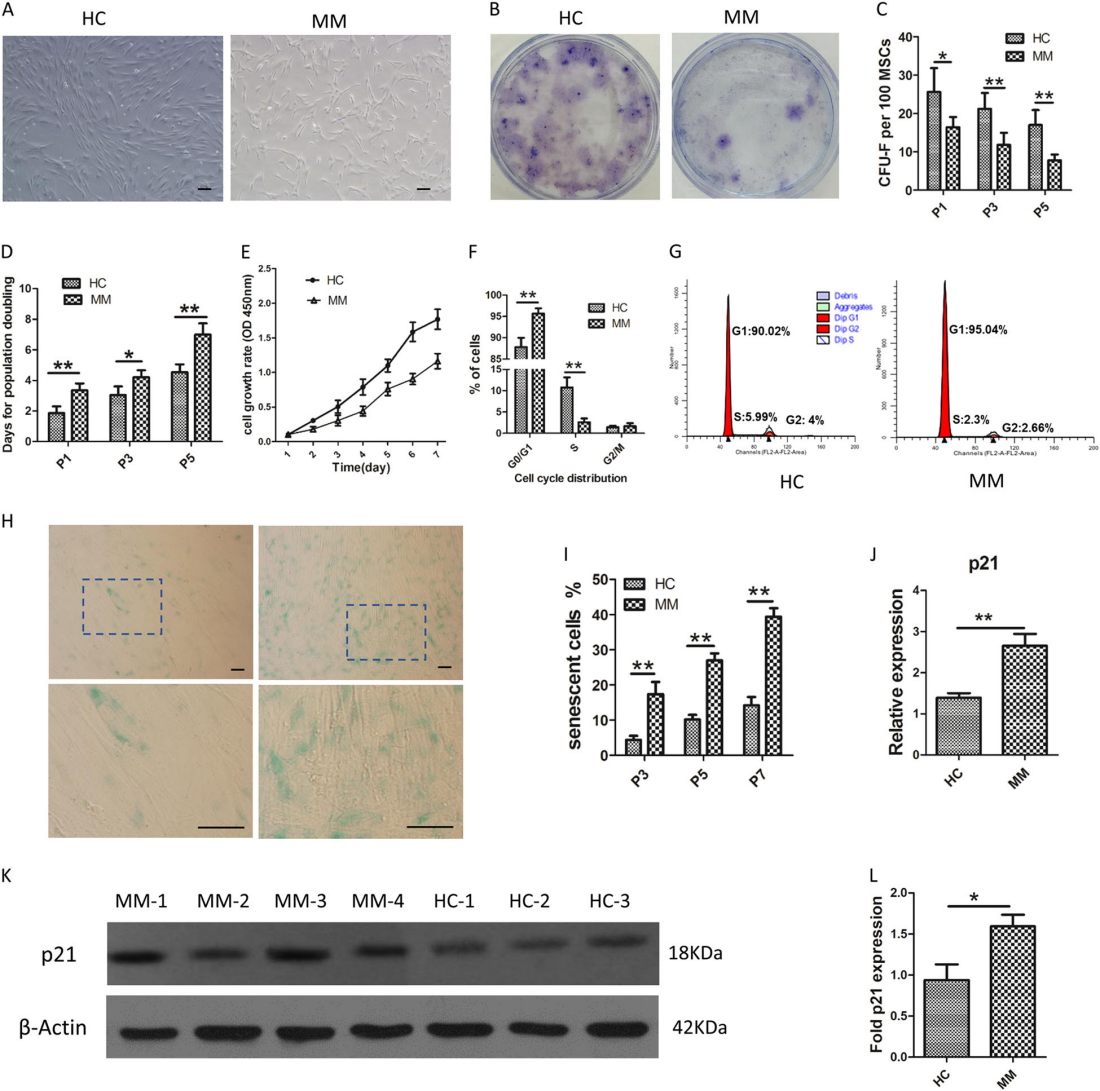
Senescent features of BMMSCs from patients with MM. **a** HC-MSCs were characterized by spindle-like morphology, while MM-MSCs were larger and irregular (40× magnification). **b** Typical colonies at the third passage in six-well plates are exhibited. **c** MM-MSCs showed degressive cumulative CFU-F numbers at passages 1, 3, and 5 in comparison with HC-MSCs. **d** Doubling times of MSCs at passages 1, 3, and 5 were obviously higher in MM patients (*n* = 15) than of control group(*n* = 10). **e** Cell proliferation ability was determined by CCK-8 assay. After the culture period of 7 days, the count of living cells in accordance with the optical density from MM groups were growing more slowly than that from the control group (*n* = 4). **f**, **g** Cell cycle distribution of MSC was determined by flow cytometric analysis. We observed a reduction in S phase and an increase in G_0_/G_1_ phase for MM-MSC (*n* = 10) compared to HC-MSCs(*n* = 5). **h**, **i** SA-β-gal was performed to identify the senescent MSCs. The amount of SA-β-gal-positive cells was significantly elevated in MM-MSCs (*n* = 15) at each time point detection. **j**–**l** Expression of senescence-related gene *p21* increased in MM-MSC (*n* = 46), as determined by RT-PCR and western blot. The results are expressed as means ± SD. Compared with HC-MSC, the significance was set as *p ≤ 0.05; **p ≤ 0.01

HC-MSCs were characterized by spindle-like morphology, while MM-MSCs were larger and irregular (Fig. 1a). The clonogenic ability of the MSCs were identified by colony forming unit fibroblast (CFU-F). MM-MSCs showed degressive cumulative CFU-F numbers at passages 1, 3, and 5 in comparison with HC-MSCs (*p* < 0.05, Fig. 1b, c). In addition, the doubling times of MSCs at passages 1, 3, and 5 were obviously higher in MM patients than of control group (Fig. 1d). Cell proliferation ability was determined by CCK-8 assay. After the culture period of 7 days, the count of living cells in accordance with the optical density from MM groups were growing more slowly than that from the control group (Fig. 1e). To demonstrate whether the reductive MM-MSCs growth was caused by weak proliferation or intensive apoptosis, the distributed population of cells in each cell cycle phase was evaluated. No Sub-G_0_ peak, in accordance with hypodiploid apoptotic cells, was existed in the BMMSC cultures (<1%). Nevertheless, the percentage of MM-MSCs in G_0_/G_1_ phase was increased to 95.66 ± 1.26% (vs 87.78 ± 2.20%; **p < 0.01), while the percentage of MM-MSCs in S phase was reduced to 2.64 ± 0.87% (vs 10.77 ± 2.39%; **p < 0.05) when compared with that of HC-MSCs (Fig. 1f, g).

SA-β-gal was performed to identify the senescent MSCs. The number of SA-β-gal-positive cells was significantly elevated in MM-MSCs at each time point detection (Fig. 1h, i). From passage 3, the median percentage of senescent cells in HC-MSCs accounted for 4.4% of all HC-MSCs. At a cut off value of 4.4%, increased senescence was observed in 86.67% (26/30) of MM-MSCs. Next, we measured the expression of *p21*, which is involved in the regulation of cell senescence. Using RT-PCR and western blotting, we found an increase in *p21* in MM-MSCs in comparison with the control group (Fig. 1j–l). Moreover, the level of *p21* expression increased in MM-MSCs (S-MM-MSCs, SA-β-gal-positive cells ≥4.4%) when compared with non-senescent MM-MSCs (NS-MM-MSCs, SA-β-gal-positive cells <4.4%).

In accordance with the above phenomena, primary MSCs (CD271^+^) from MM patients also exhibited raised cell senescence, which was displayed by an obviously increased amount of SA-β-gal positive cells and increased *p21* expression level, in comparison with the healthy control group. The collective data indicate that the proliferation capacity decreased and the senescence increased in MSCs from MM patients.

### Senescent MM-MSCs exhibited decreased differentiation

**Fig. 2:**
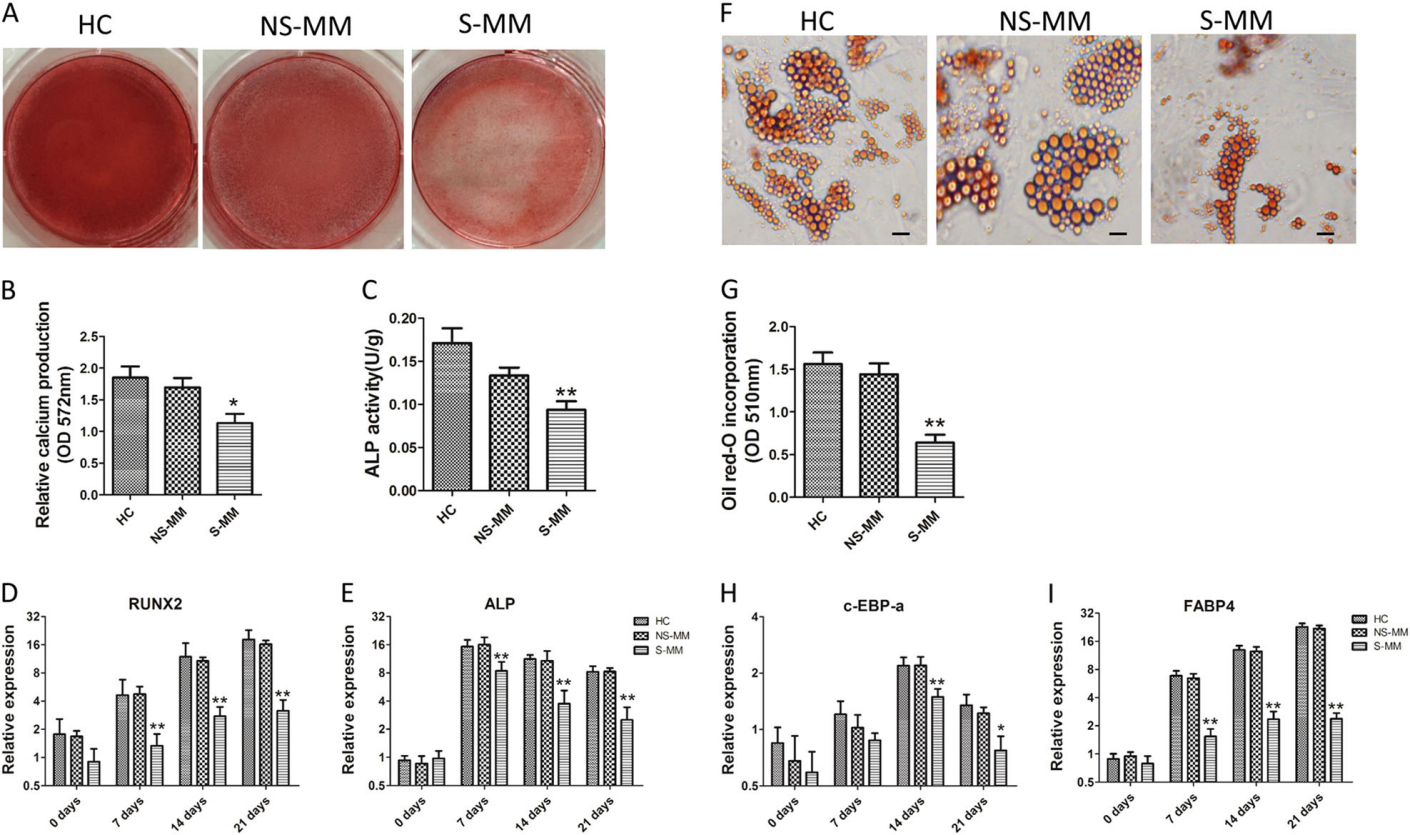
Senescent MM-MSCs (S-MM-MSC) exhibited decreased differentiation. **a**–**c** Mineralization analysis and ALP activity measurements. The mineralization was visualized by Alizarin Red S staining at 21 days. ALP activity was measured at 7 days after osteogenic differentiation using an alkaline phosphatase activity kit. In comparison with HC-MSCs and NS-MM-MSCs, senescent MM-MSC showed significantly reduced osteogenic differentiation potential, which is indicated by the results of mineralization analysis and activated ALP evaluation. **d**, **e** The mRNA expressions of RUNX2 and ALP, were notably decreased in senescent MM-MSCs undergoing osteoblastic differentiation at 0, 7, 14, and 21 days. **f**, **g** The ability of adipogenic differentiation was identified by Oil Red-O staining, and it was increased significantly in HC-MSCs than that in senescent MM-MSCs. **h**, **i** The differences of the adipogenic differentiation capability between HC-MSCs and senescent MM-MSCs were also revealed in the expressions of FABP4 and C/EBPa mRNAs.The results are expressed as means ± SD. Compared with HC-MSC, the significance was set as *p ≤ 0.05; **p ≤ 0.01

On account of that cell dysfunction is relevant to cell senescence, we identified the ability of senescent MM-MSC and HC-MSCs to differentiate and to promote tumor cell proliferation. The osteoblastic and adipogenic differentiation capabilities of MSCs were assessed by immunohistochemical method and associated genes expression analysis. In comparison with HC-MSCs and NS-MM-MSCs, senescent MM-MSC showed significantly reduced osteogenic differentiation potential, which is indicated by the results of mineralization analysis and activated ALP evaluation (Fig. 2a–c). In accordance with the immunohistochemical staining analysis, the mRNA expressions of *RUNX2* and *ALP*, which play crucial role in the process of osteoblastic differentiation, were notably decreased in senescent MM-MSCs undergoing osteoblastic differentiation (Fig. 2d, e). The ability of adipogenic differentiation was identified by Oil Red-O staining, and it was increased significantly in HC-MSCs than that in senescent MM-MSCs (**p < 0.01; Fig. 2f, g). The differences of the adipogenic differentiation capability between HC-MSCs and senescent MM-MSCs were also revealed in the expressions of FABP4 and C/EBPa mRNAs (Fig. 2h, i).

In addition, there exists a significant difference in these two group of MM patients. The patients with high β-gal staining MM-MSCs (S-MM) showed different disease progression compared to those with low β-gal staining MM-MSCs(NS-MM). Progression free-survival (PFS) was calculated from the date of the diagnosis to the date of progression. Patients with less than 4.4% SA-β-gal-positive cells at diagnosis, had significantly longer PFS (mean of 29 v 11 months; *p* = 0.014; Supplementary Figure S1) than patients with more than 4.4% SA-β-gal-positive cells.

### Low expression of Dicer1 was exhibited in MM-MSCs, and MM cells induced senescence in HC-MSCs

**Fig. 3:**
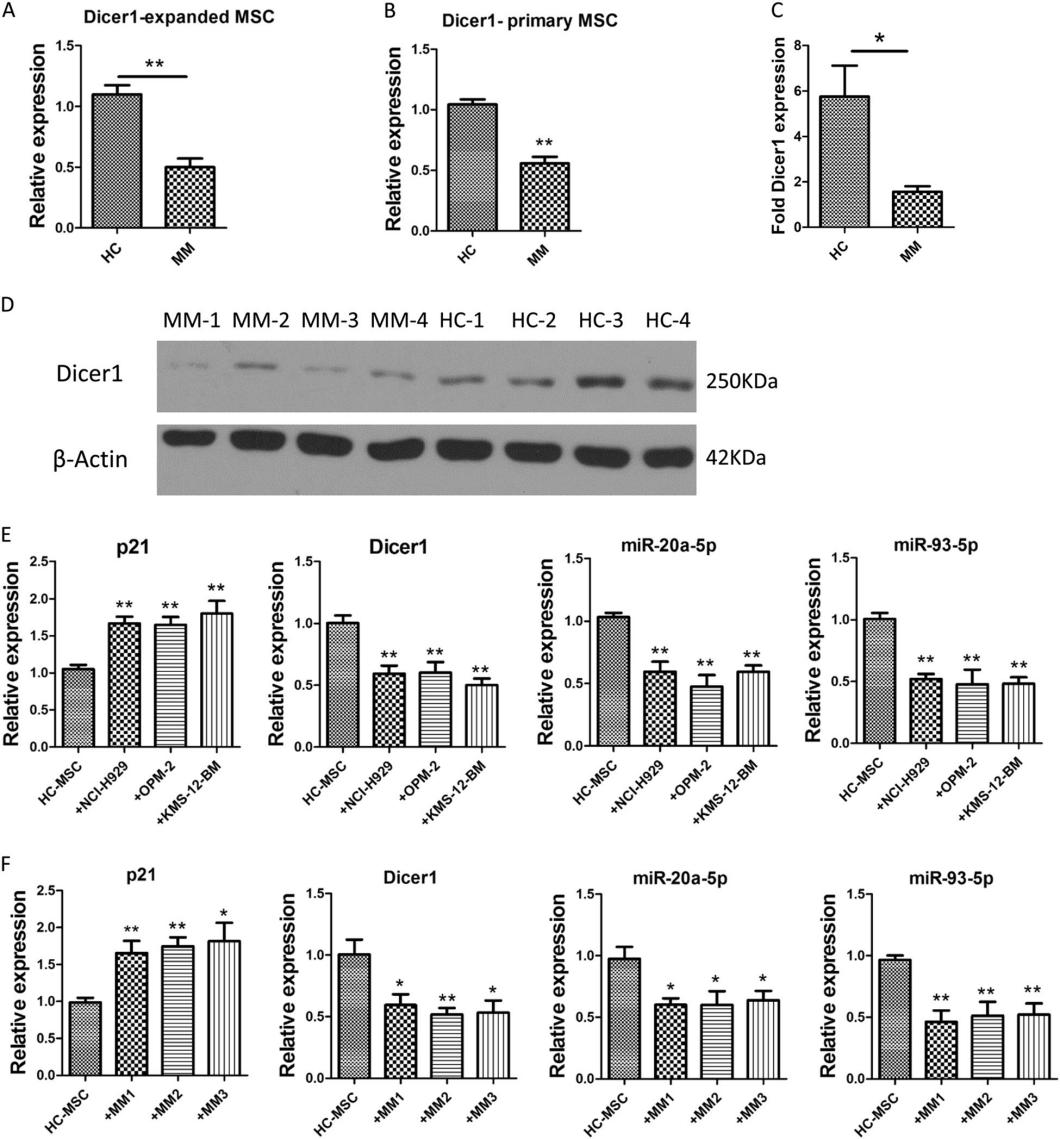
Low expression of Dicer1 in MM-MSCs and myeloma cells induce cell senescence in HC-MSC. **a** The mRNA expression of Dicer1 in expanded MM-MSC (*n* = 46) and HC-MSC (*n* = 18). **b** The expression of Dicer1 in primary MM-MSC (*n* = 18) and primary HC-MSC (*n* = 10). **c**, **d** Protein levels of Dicer1 in MM-MSC (*n* = 4) and HC-MSC (*n* = 4). **e**, **f** HC-MSC (*n* = 4) were co-cultured with HMCLs: NCI-H929, OPM-2, and KMS-12-BM (**e**), or with primary CD138^+^ MM tumors cells from three MM patients (**f**) for 24 h. Relative mRNA expression levels of *p21*, Dicer1, miR-93, and miR-20a in MSC after co-cultured with Myeloma cells. The results are expressed as means ± SD. Compared with controls, the significance was set as *p ≤ 0.05; **p ≤ 0.01

The attenuation of Dicer1 resulted in rapid cell senescence in a variety of cells. We examined the level of Dicer1 in in vitro expanded and primary (CD271^+^) MSCs derived from MM patients and healthy controls. Results from RT-PCR and western blot exhibited that the expression of Dicer1 was declined in MM-MSCs (Fig. 3a–d).

To determine whether myeloma cells could affect MSCs, HC-MSCs were co-cultured with myeloma cell lines (such as NCI-H929, OPM-2, and KMS-12-BM) or with primary CD138^+^ tumors cells from three MM patients for 24 h and the expressions of Dicer1, *p21*, miR-93 and miR-20a was determined by RT-PCR. We used trans-well inserts with 1-μm pores. MM cell lines or MM primary cells were cultured in the upper chamber of the inserts. We observed that myeloma cells could significantly decrease the expression of Dicer1, *p21*, miR-93 and miR-20a in HC-MSCs (Fig. 3e, f). The cells in the lower chamber were stained by SA-β-gal assay and results confirm that the cells were undergoing senescence. A few of staining were identified in the control MSC, while SA-β-gal activity was clearly obvious in the co-cultured MSC (data not shown).

### Knockdown of Dicer1 induced senescence and inhibited differentiation of HC-MSCs

**Fig. 4:**
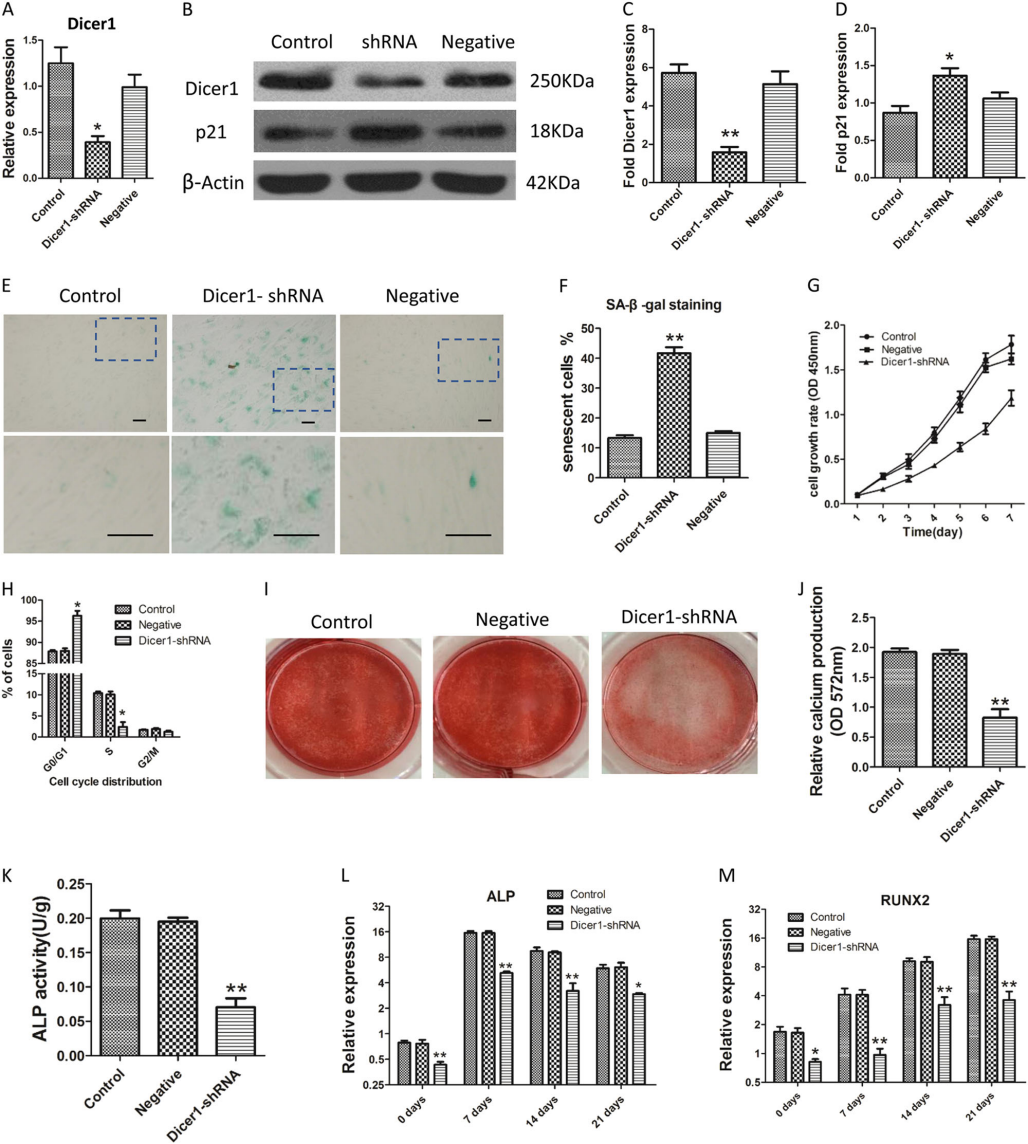
Knockdown of Dicer1 induced senescence and inhibited osteogenic differentiation of HC-MSCs. **a**–**d** The expressions of Dicer1 mRNA and protein were dramatically decreased compared to that of the control group. And the protein expression of *p21* increased. **e** Representative micrographs after SA-β-gal staining of Dicer1-KD MSC (shRNA), negative MSC (transfected with control lentiviruses) and control-MSC (HC-MSC without transfection) (100× magnification). **f** One hundred MSC per sample were counted using light microscopy, and the percentages of SA-β-gal-positive cells were determined. The average of three replicates is displayed. **g** The proliferation of MSCs treated with Dicer1 knockdown (KD) was obviously inhibited in comparison with either control MSCs or the negative group. **h** Cell cycle analysis of Dicer1-KD MSC by flow cytometric analysis. Dicer1 KD caused an increasing proportion of cells in the G1 phase and a decrease of those in the S phase without inducing apoptosis. **i** After 21 days of osteogenic induction, Alizarin red S staining was performed to visualize osteogenic differentiation. Representative original images of BMMSCs derived from control-MSC (HC-MSC without transfection), negative MSC (transfected with control lentiviruses), Dicer1-KD MSC are shown. **j** Relative calcium production (OD 572 nm) by Dicer1-KD MSC, was significantly lower after 21 days of differentiation as compared with controls. **k** The ALP activity of Dicer1-KD MSC was significantly lower than that of controls after 3 days culturing in osteogenic medium (OM). **l**, **m** Relative *RUNX2* and *ALP* mRNA expression levels. The average of three replicates is displayed. Compared with controls, the significance was set as * *p* ≤ 0.05; ** *p* ≤ 0.01

We then constructed a recombinant lentivirus to decrease the expression of Dicer1 to further study the biological effect of Dicer1 on MSC senescence. It is displayed in Fig. 4a–d that the expressions of Dicer1 mRNA and protein were dramatically decreased compared to that of the control group. The proliferation of MSCs treated with Dicer1 knockdown (KD) was obviously inhibited in comparison with either control MSCs or the negative group (Fig. 4g).

To demonstrate whether the cells were growing senescent, Dicer1-KD MSCs or control groups were identified once they reached confluence. On the 14th day, Dicer1-KD MSCs stopped division and exhibited a large, flattened shape. Cells were collected and replanted in fresh medium at higher or lower densities and the proliferation cannot be promoted. To identify that Dicer1-KD MSCs were becoming senescence, cells were then stained by SA-β-gal assay. Light staining was determined in the control and negative groups, while SA-β-gal activity was quite clear in the Dicer1-KD MSCs (Fig. 4e). Exceeding 40% of the Dicer1-KD MSCs were growing senescence after 7 days, in comparison with less than 14% of the control and negative groups (Fig. 4f). The above results reveal that down-regulation of Dicer1 is effective for MSC senescence induction. Furthermore, Dicer1 KD caused an increasing proportion of cells in the G_1_ phase and a decrease of those in the S phase without inducing apoptosis (Fig. 4h).

We measured the biological role of Dicer1-KD on the capability of MSCs to differentiate into osteogenic and adipogenic cells. Dicer1-KD MSCs were treated with osteogenic induction and exhibited decreased mineralization when stained with Alizarin red (Fig. 4i). Furthermore, they showed a reduction in ALP activity compared with negative MSCs and control cells (**p < 0.01) (Fig. 4j, k). In the process of osteogenic induction, mRNA levels of ALP and RUNX2 were elevated significantly in the control MSCs. On the contrary, the expressions of these mRNAs increased mildly in Dicer1-KD MSCs (Fig. 4l, m).

We also assessed the ability of MSCs differentiating into an adipogenic lineage treated with knockdown of Dicer1. On Day 21, Oil red-O-stained cultures revealed that excessive 91% of control MSCs could differentiate into adipocytes, exhibiting plenty of cellular lipid droplets. On the contrary, the cellular lipid amount in Dicer1-KD MSCs was rare, consisting of small droplets (Supplementary Figure S2A). In the adipogenic inductive process, the expressions of C/EBPa and FABP4 were significantly elevated. After 14 days, control MSCs showed an obvious raise in C/EBPa and FABP4 in comparison with undifferentiated cells. On the contrary, Dicer1-KD MSC showed only a slight increase in mRNA expressions, with a 1.64-fold raise in C/EBPa and a 0.64-fold raise in FABP4 when in comparison with undifferentiated cells (Supplementary Figure S2B, C).

### Down-regulation of Dicer1 in MSCs stimulated the proliferation and reduced apoptosis of MM cells

**Fig. 5:**
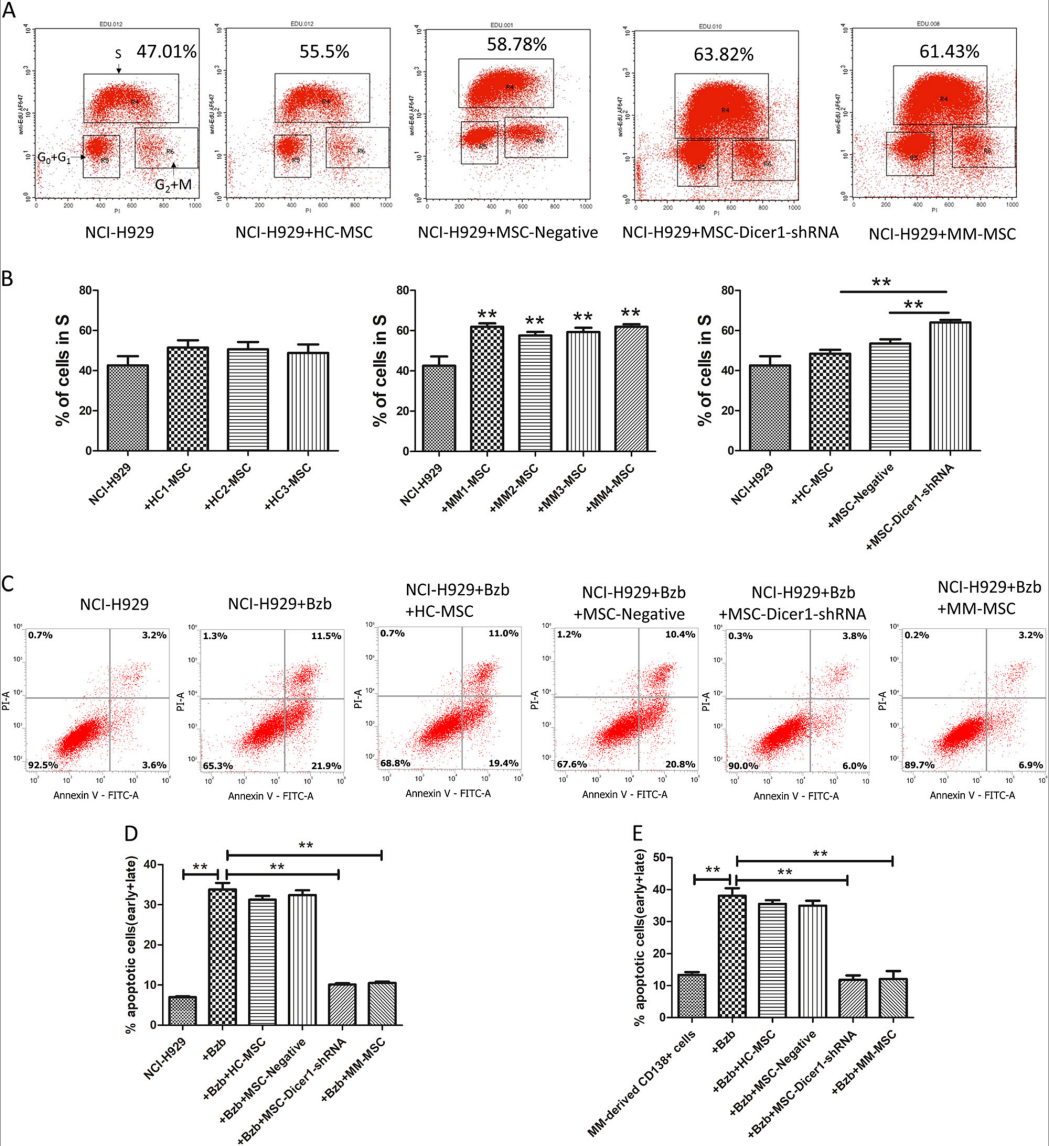
Down-regulation of Dicer1 in MSCs stimulated MM cell proliferation and protected MM cells against apoptosis in vitro. **a** NCI-H929 were co-cultured with MSCs: control-MSC (HC-MSC without transfection), negative MSC (transfected with control lentiviruses), Dicer1-KD MSC (shRNA), and MM-MSC for 24 h. Cell cycle behavior of H929 was assessed by Click-iT Edu Flow Cytometry Assay. Fluorescence-activated cell analysis plots from one representative experiment showing the cell cycle plots for cultured alone MM cell lines(H929) or with MSCs as indicated. The location of cells in different phases of the cell cycle is indicated in the left histogram, and the percentage of cells in S is given for each condition. **b** Summary of results from separate experiments. H929 cultured alone (control), or co-cultured with HC-MSCs from three healthy controls(H929 + HC-1-3MSC), or co-cultured with MM-MSCs from four different patient samples (H929 + MM1-4-MSC), or co-cultured with negative MSC (transfected with control lentiviruses), Dicer1-KD MSC (shRNA) for 24 h were assessed for the proportion of cells in S by Click-iT Edu Flow Cytometry Assay. The results are expressed as means ± SD. The average of three replicates is displayed. Compared with controls, the significance was set as *p ≤ 0.05; **p ≤ 0.01. **c**–**e** MSCs were cocultured in complete growth medium with MM cells (NCI-H929 or CD138^+^ MM cells) for 48 h at a ratio of 1:10 (MSCs/MM cells). As measured by AnnexinV/PI staining, MM-MSCs could protect against Bortezomib-induced apoptosis at a ratio of 1:10 (MSCs/MM cells). Similarly, MSC-Dicer1-shRNA can also protect Bortezomib-induced apoptosis of MM cells. The results are expressed as means ± SD. The average of three replicates is displayed. Compared with controls, the significance was set as *p ≤ 0.05; **p ≤ 0.01

Dicer1 knockdown increased the tumor-supporting properties of MSCs. NCI-H929 cells were co-cultured with the following MSCs: control-MSCs (HC-MSCs without transfection), negative MSCs (transfected with control lentiviruses), Dicer1-KD MSCs (shRNA), and MM-MSCs for 24 h. Cell cycle behavior of NCI-H929 cells was identified by Click-iT Edu Flow Cytometry Assay. Fluorescence-activated cell analysis plots from one representative experiment showing the cell cycle plots for monocultured MM cell lines (NCI-H929) or with MSCs as indicated. The location of cells in different phases of cell cycle is shown in the left histogram, and the percentage of cells in S is obtained for each condition (Fig. 5a). The results of the Edu assay showed that Dicer1-KD MSCs and MM-MSC promoted myeloma cell proliferation, and that the proportion of S phase cells was increased compared with that in the HC-MSC or MSC-Negative groups (Fig. 5a, b).

Relapse and chemo resistance are the main features of the clinical course of MM, and it is well-known that the bone marrow microenvironment protects MM cells against chemotherapy. Here we examined whether MM-MSCs could protect MM cells against chemotherapy-induced apoptosis. Bortezomib is a clinically available proteasome inhibitor that is currently among the most potent chemotherapeutic drugs used in the treatment of MM. When the human MM cell line NCI-H929 or MM-derived CD138^+^cells were cultured in complete medium with 5 nM Bortezomib for 48 h, approximately 33.4% apoptotic cells (early and late) were observed. However, in the presence of MSC-Dicer1-shRNA and MM-MSC, the proportion of apoptotic cells significantly decreased to 9.8% and 10.1%, respectively (Fig. 5c–e).

MSCs interact with MM cells, releasing cytokines which promote the viability of MM cells. Variational chemokine levels could cause the promoted tumor supporting effect of MM-MSCs. The expression of cytokines was then detected in MM-MSCs and they showed an increased expression of GDF-15 (growth differentiation factor-15), DKK-1 (the Inhibitor of the Wnt signaling pathway--Dickkopf1), and IL-6 (interleukin-6) compared with control MSCs. However, the level of SDF-1(stromal cell derived factor 1) was significantly under-expressed in MM-MSCs compared to HC-MSCs. Cytokines were evaluated by real-time RT-PCR and ELISA. (Supplementary Figure S3).

### Upregulation of Dicer1 reversed the senescent features of MM-MSCs and restored their differentiative capacity

**Fig. 6:**
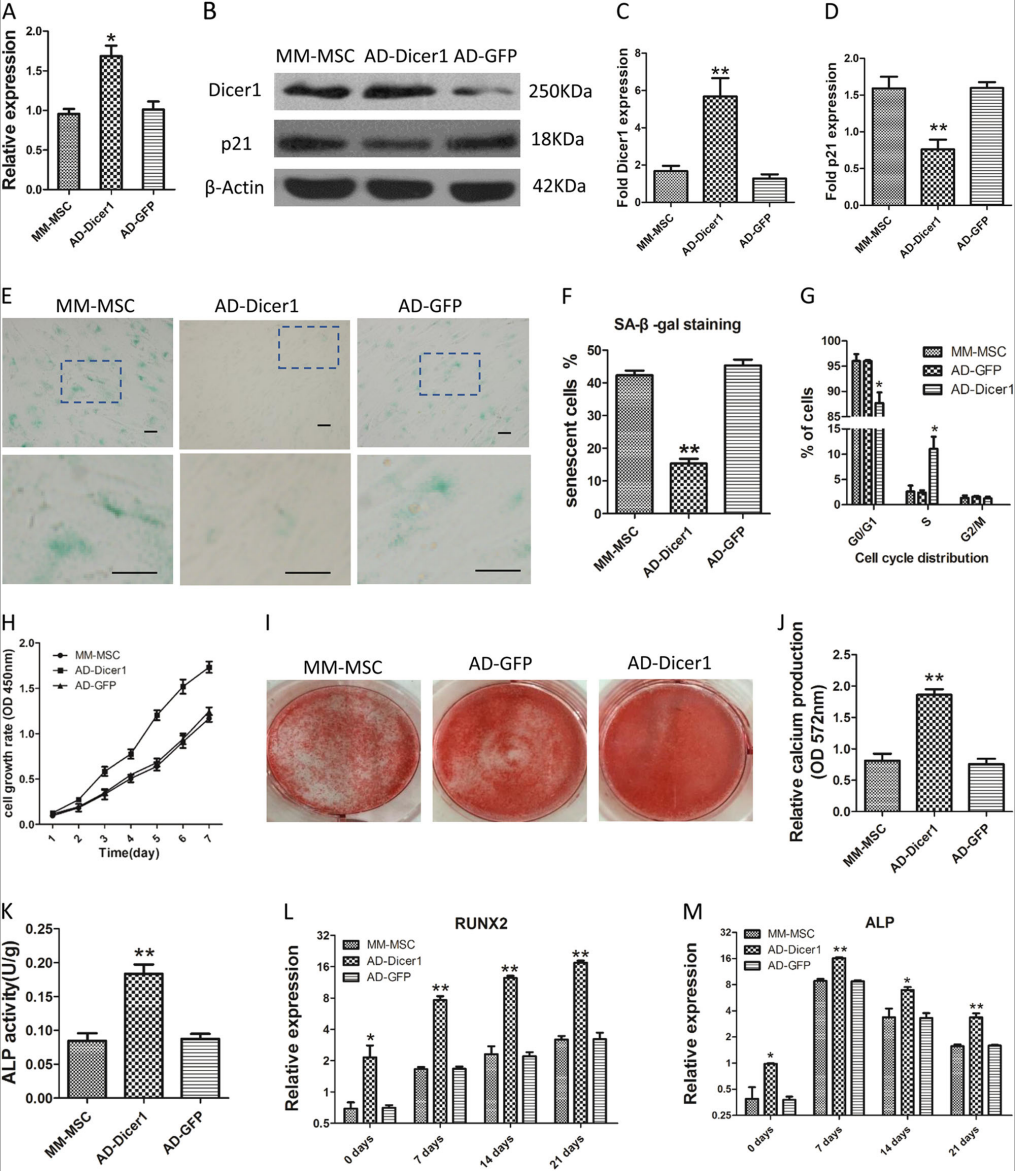
Upregulation of Dicer1 reversed the senescent features and osteogenic differentiation of MM-MSCs. **a**–**d** The expressions of Dicer1 mRNA and protein were dramatically increased after adenovirus transfection. And the protein expression of *p21* decreased. **e** Representative micrographs after SA-β-gal staining of control MSC (MM-MSC without transfection), AD-Dicer1 MSC (MM-MSC transfected with Dicer1 adenovirus lentiviruses) and AD-GFP (MM-MSC transfected with control lentiviruses) (100× magnification). **f** The percentages of SA-β-gal-positive cells. **g** Cell cycle analysis of Dicer1-KD MSC by flow cytometric analysis. Dicer1 AD caused an increasing proportion of cells in the S phase and a decrease of those in the G1 phase. **h** MM-MSCs transfected with AD-Dicer1 proliferate more quickly than either MM-MSCs or MM-MSCs transfected with AD-GFP. **i** Typical images after Alizarin Red S staining on day 21 of osteogenic differentiation. **j** Relative calcium production (OD 572 nm) by AD-Dicer1 MSC, was significantly higher after 21 days of differentiation as compared with controls. **k** The ALP activity of AD-Dicer1 MSC was significantly increased after 3 days osteogenic differentiation. **l**, **m** Relative RUNX2 and ALP mRNA expression levels. The results are expressed as means ± SD. The average of three replicates is displayed. Compared with controls, the significance was set as *p ≤ 0.05, ** *p* ≤ 0.01

In the following experiment, we performed MM-MSC transfected with an adenovirus carrying the Dicer1 gene (AD-Dicer1) or green fluorescent protein (AD-GFP). The mRNA and protein expressions of Dicer1 was significantly elevated in AD-Dicer1 MSCs (Fig. 6a–d) levels. It was shown that MM-MSCs transfected with AD-Dicer1 proliferate more quickly than either MM-MSCs or MM-MSCs transfected with adenovirus carrying green fluorescent protein (AD-GFP) (Fig. 6h). There were no evident SA-β-gal-positive cells in MM-MSCs with Dicer1 overexpression (Fig. 6e, f). Furthermore, overexpression of Dicer1 gave rise to an increasing number of cells in the S phase and decreased in the G1 phase (Fig. 6g). Furthermore, AD-Dicer1 MSCs showed increasing osteoblastic (Fig. 6i–m) and adipogenic differentiation ability (Supplementary Figure S4) in comparison with MSCs which was treated with GFP vectors transfection.

### MiR-17 family members participated in Dicer1 KD-induced senescence

**Fig. 7:**
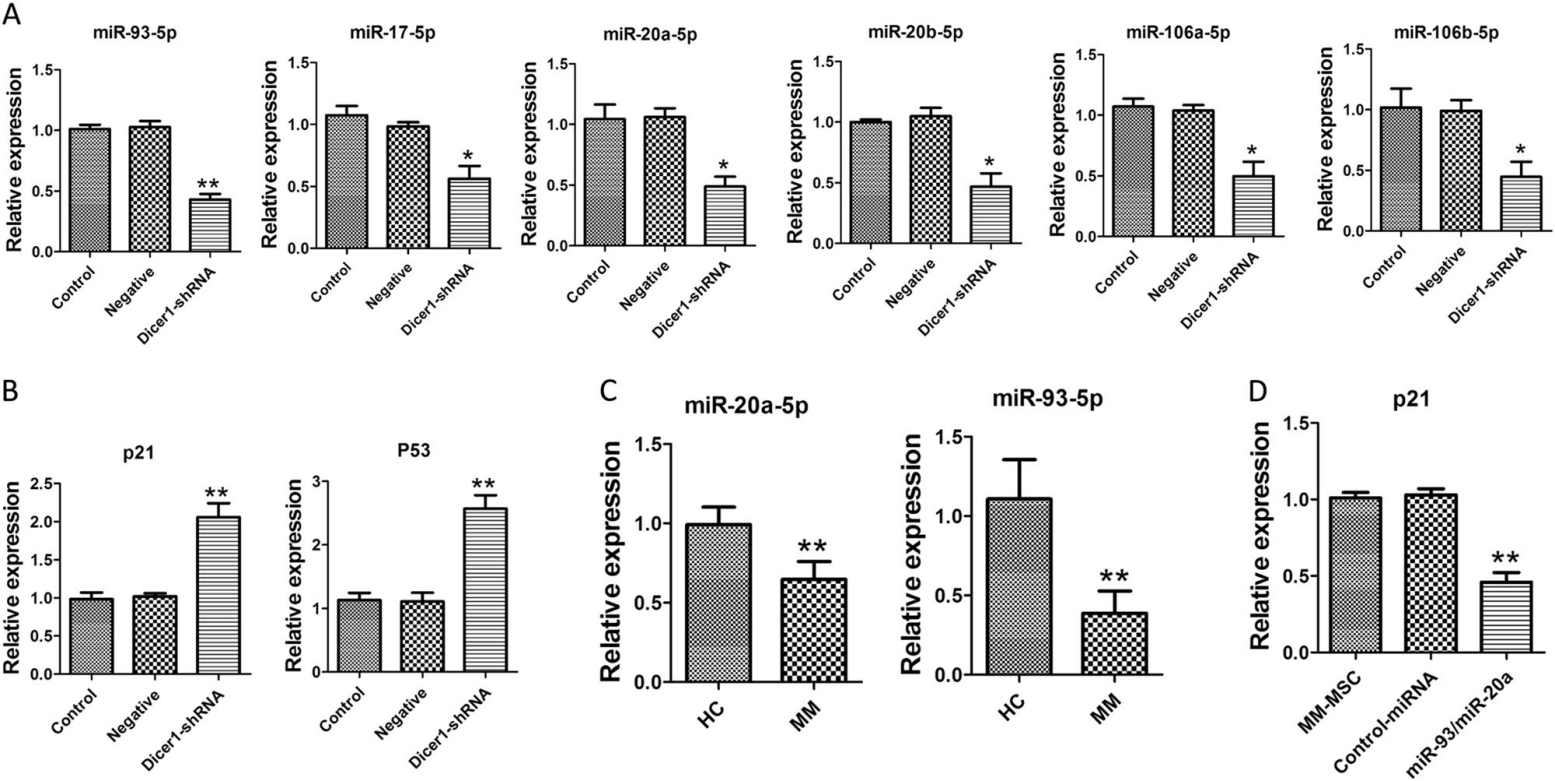
MiR-17 family members participated in Dicer1 KD-induced senescence. **a** The expression of miR-17 family of Dicer1-KD MSC (shRNA), negative MSC and control-MSC were detected by Real-time PCR. Decreased expressions of miR-93 and miR-20a was also appeared in MM-MSCs when compared with HC-MSCs. **b** Relative mRNA expression levels of p21 and p53 in Dicer1-KD MSC. **c** Decreased expressions of miR-93 and miR-20a was also appeared in MM-MSCs (*n* = 36) when compared with HC-MSCs (*n* = 18). **d** MM-MSCs were transfected with miR-93 and miR-20a harboring lentiviruses, and p21 levels after 48 h transfection was also detected. These experiments were performed three times. Compared with controls, the significance was set as *p ≤ 0.05; **p ≤ 0.01

To identify whether lower expression of Dicer1 in MSC could show effect on miRNA biogenesis and take part in cell senescence, we detected a number of specific miRNAs which possibly target senescence-associated molecules, such as p21. miRBase, PicTar and Targetscans were performed to screen targets. miR-20a expression was significantly decreased in Dicer1-KD MSCs in comparison with both control MSCs and the negative group (Fig. 7a,b). Decreased expressions of miR-93 and miR-20a was also appeared in MM-MSCs when compared with HC-MSCs. To demonstrate that p21 is a target of miR-93 and miR-20a in MM-MSCs, studies involving miR-93/miR-20a up-regulation were employed. MM-MSCs were transfected with miR-93 and miR-20a harboring lentiviruses, and *p21* levels after 48 h transfection was also detected. In three analyzed samples of MM-MSCs, the overexpression led to decreased *p21* expression (Fig. 7d) compared with that in cells transfected with scrambled control lentivirus.

## Discussion

MM cells are believed to have a reciprocal interaction with the surrounding tumor microenvironment, which consists of endothelial cells, osteoclasts, fibroblasts, macrophages, and other cells. These cells play a crucial part in support of the proliferation, survival, chemo-resistance, and migration of MM cells^5^. A lot of studies showed that the role of the BM microenvironment in cytokines and growth factors secretion that could promote MM pathological mechanism and therefore take part in disease progression. After co-culture with MM cells, normal MSCs showed a phenotype as similar with that of MM-MSCs^25^, demonstrating that MM cells induce this phenotypic change, either by immediate contact or by soluble elements^3, 26^. The current results indicated a new mechanism by which BMMSCs act as an oncogenic factor in MM.

In the present study, we first confirmed that MM-MSCs were more likely to be senescent than HC-MSCs. Although MSCs from all MM patients were expanded successfully in vitro, MM-MSCs showed a reduced expansion potential when compared to HC-MSCs. These observations are in line with the findings of several previously published studies, but conflict with other research in which MM-MSCs had normal proliferative characteristics. The typical features of cellular senescence include enlarged morphology, decreased cellular proliferation, and increased SA-β-gal expression. Exactly as expected, most of the cultured MM-MSCs were larger, flatter, irregular and expressed observably higher p21 (the senescence-related molecule). It is clear that the amount of SA-β-gal positive cells was significantly higher in MM-MSCs than HC-MSCs. Our findings are in agreement with previous studies, but these studies did not detail the mechanisms and functional changes associated with senescence in the pathogenesis of MM.

The characteristics of senescent MSCs include changes in cellular functions. We demonstrated that senescent MM-MSCs displayed decreasing differentiation capability and increased tumor supporting ability. The depressed osteogenic and adipogenic potentials in senescent MM-MSCs have been verified in vitro by quantitative and continuous detection. In particular, some cell surface molecules and subsequent downstream signaling pathways take part in the regulation of MM-related bone destroying process, in which the balance of bone resorption and formation are no longer acts as a result of the increasing activity of osteoclasts, but rather the osteoblast activity is decreased, leading to an uncoupled or severely imbalanced bone remodeling process. In addition, our previous study showed that MM patients exhibit an impairment of osteogenic differentiation of BMMSCs as compared with healthy controls^27^. In the current study, knockdown of Dicer1 in HC-MSCs decreased the osteogenic differentiation of MSCs. Moreover, MM-MSCs exhibited an increased tumor-supporting capacity. Thus, the senile MSCs could be a promoting element in favoring myeloma cell growth of patients with MM. Furthermore, knockdown of Dicer1 in HC-MSCs promoted cellular senescence and tumor-supporting capacities, while decreasing the differentiation of MSCs. We confirmed that MM-MSCs and Dicer1-KD MSCs increased the proportions of S phase myeloma cells. In accordance with our findings, some studies showed that MM-MSCs displayed tumor-supporting capability compared with healthy controls. However, some of the previous reports demonstrated that MSCs possibly have inhibition capability. For example, Ramasamy et al. reported that MSCs act directly on tumor cells, suppressing proliferation and inducing apoptosis in tumor cells^28^. Whether MSCs promote or inhibit myeloma cell growth remains controversial. The diversities in these findings are probably due to the different origin of MSCs, cellular populations and experimental protocols in the respective studies.

In recent years, miRNAs, a pivotal group of moderating factors, have been deemed to be prominent regulators of cellular senescence^18, 19^. It is reported that Dicer1 is an RNAse III endonuclease responsible for microRNA biogenesis^22^. Furthermore, our preliminary findings showed that the mRNA and protein levels of Dicer1 in MSCs isolated from MDS patients was significantly decreased compared with MSCs from health controls^17^. On the basis of these findings, we evaluated Dicer1 expression in MM-MSCs. It was demonstrated that the expression of Dicer1 decreased in primary and expanded MM-MSCs. In the current study, we investigated the effects of a lower expression of Dicer1 in MM-MSCs by generating a stable knockdown model. Our study is mainly concerned with the biological function of Dicer1 in cell senescence, differentiation and tumor support of Dicer1-KD MSC expedited cellar senescence, which manifested as flattened cell morphology, increased SA-β-gal positive incidence and a decreased percentage of cells in S phase. Experiments involving Dicer1 overexpression further verified these results. Upregulation of Dicer1 expression in MM-MSC reversed cellular senescence and promoted differentiation. Under-expression of Dicer1 interferes with the biogenesis of miRNA, which is relevant to cellular senescence in endothelial cells. However, the role of miRNA in the senescence of BMMSCs remains ambiguous. Senescent cells generally exhibit increased expression of cell cycle inhibiting factor such as p53/p21, which could be adjusted by miRNA^29–31^. In our current study, decreased expression of the miR-17 family (miR-106a/b, miR-17-5p, miR-20a/b, and miR-93) was determined to be a critical factor responsible for elevated *p21* expression in Dicer1-KD MSC. Actually, miRNAs in the miR-17 family have been identified as regulators of cell cycle through targeting p21 in many other studies^32–34^. It was demonstrated that a lower of expression of the miR-17 family could give rise to the Dicer1-KD-induced cell senescence in MSCs in our present and previous studies. Lately, substantial miRNA expression was reported to be downregulated in MM-MSCs, demonstrating that reduced miRNA expression probably be associated with the pathogenic mechanism of MM. Our result showed that expression of miR-93 and miR-20a remarkably decreased in MM-MSCs. Moreover, overexpression of miR-93/ miR-20a reversed cellular senescence in MM-MSCs through targeting p21. It was confirmed that miRNA showed a critical effect on increasing cell senescence of MM-MSCs.

Dicer1-KD MSCs displayed diminished osteogenic differentiation potential, characterized by the reduction in the expression of osteoblastic maker genes, calcium deposits and ALP activity, which was consistent with previous studies. Furthermore, upregulation of Dicer1 increased the impaired osteoblastic differentiation capability of MM-MSCs. Recently, many studies demonstrated that osteolytic bone lesions of MM patients resulted from the abnormal osteoblastic function in patients^6, 35–37^. Our study reported that the reduction of miRNA processing induced by Dicer1-KD was effective to immensely decrease the differentiation ability of MSCs, which indicated that exact level of mature miRNAs is important for MSC osteoblast differentiation. Most importantly, it provides a new perspective for the study of bone lesions in MM.

The senescent MSCs not only exhibited an impaired differentiation potential but also an enhancement in their support of tumor growth. So far, knowledge concerning the effect of MSC senescence in tumor growth support is restricted. In our results, in addition to the classic features of cell senescence, Dicer1-KD MSCs exhibited a significant increase in their capacity to support myeloma cells. Furthermore, BMMSCs derived from patients with MM were found to deliver the cytokine IL-6, which is known to promote tumor formation and progression. The tumor microenvironment-derived GDF15 was also able to significantly increase cell survival in primary MM cells.

## Conclusions

In this study, we explored the feature and molecular mechanism of MSCs senescence in MM patients. Dicer1 plays a crucial role in the senescent process of MM-MSCs by regulating miRNA. Senescent MM-MSCs have a decreased differentiation potential and an increased tumor-supporting capacity. Therefore, much attention should be paid to the therapeutic exploitation of MSCs in bone lesion diseases and tumor progression.

## Methods

### Patients

A total of forty-six patients with newly diagnosed MM (*n* = 46) were enrolled between January 2015 and June 2017 in this study (Table 1). All experiments were approved by the Ethics Committee of Shanghai Jiao tong University Affiliated Sixth People’s Hospital (Shanghai, China) and written informed consent was obtained from all subjects. Patients were diagnosed according to the criteria recently defined by the International Myeloma Working Group^38^, and were staged according to the criteria of ISS^39^. For the patients enrolled, they were all uniformly treated with bortezomib and dexamethasone. The median follow-up was 24 months (rang 9–38 months). The bone marrow was extracted for experiments before the treatment start. Eighteen healthy controls with the median age of 66 years (age range, 46–79 years) were analyzed in our study and they were matched for gender and age.

**Table 1:** Clinical characteristics of patients with multiple myeloma

Characteristic	*N* = 46
Sex	
Male, *n* (%)	25 (54%)
Female, *n* (%)	21 (46%)
Median age, years (range)	65 (38–85)
Immunoglobulin subtype, *n* (%)	
IgG	22 (48%)
IgA	10 (22%)
Light-chain	
κ	6 (13%)
λ	2 (4%)
Non-secretory	6 (13%)
International staging system, *n* (%)	
I	22 (48%)
II	14 (30%)
III	10 (22%)

### Cell culture

Bone marrow MSCs from patients and controls were isolated, cultured and harvested. The procedure was detailed in our previous studies. Human MM cell lines NCI-H929, OPM-2, and KMS-12-BM were cultured in RPMI 1640 medium (Gibco, Grand Island, NY, USA), supplemented with 10% fetal bovine serum (Gibco), 100 units/mL penicillin, and 100 mg/mL streptomycin.

### Cell proliferation

The proliferation of BMMSCs was detected by using CCK-8 (Dojindo, Japan) and by measuring the cell doubling time from P1 to P5. To evaluate the clonogenic potential of BMMSCs, colony-forming unit fibroblast (CFU-F) at P1, P3, and P5 were counted (colonies were stained with Giemsa).

### Co-culture of BMMSCs with myeloma cells and 5-Ethynyl-2′-deoxyuridine (EdU) incorporation assay

The human multiple myeloma cell lines NCI-H929 were purchased from the American Type Culture Collection (ATCC, Manassas, VA, USA). The cells were cultured in RPMI 1640 (Gibco) medium supplemented with 10% fetal bovine serum (Gibco),100 units/mL penicillin, and 100 mg/mL streptomycin in a humidified atmosphere with 5% CO_2_ at 37 °C. NCI-H929 were co-cultured with MSCs (HC-MSCs or MM-MSC) for 24 h. We used a trans well inserts with 1-μm pores. MM cell lines or MM primary cells were cultured in the upper chamber of the inserts. (For cell cycle and proliferation analysis, cells were exposed to 10 μM EdU (Invitrogen) in culture medium for 45 min. EdU was detected with Alexa Fluor 647-azide with the use of a Click-iT EdU Flow Cytometry Assay Kit (Invitrogen). Flow cytometry was performed with a FACS Calibur flow cytometer (Becton Dickinson, SanJose, CA, USA) and Cell Quest software.

### SA-β-gal assay

BMMSCs were plated into 6 well culture-plates and maintained until 80% confluence. The cells were incubated with SA-β-gal staining solution (Beyotime, China) according to the manufacturer's instructions. Under inverted microscope, the number of SA-β-gal positive cells (blue cells) were calculated as senescent cells.

### Cell cycle analysis

Cells were fixed by precooled ethanol for at least 24 h and stained with the PI/RNase Staining Solution kit (Invitrogen, Carlsbad, CA, USA) according to the manufacturer’s directions. The cell cycle analysis was evaluated by flow cytometry and MultiCycle software.

### Cell apoptosis analysis

Apoptosis of MM cells was induced by Bortezomib (Selleck, Shanghai, China). The quantification of apoptotic cells was performed by using the AnnexinV-FITC Apoptosis Detection Kit (Invitrogen, Carlsbad, CA, USA) according to the manufacturer’s instructions. The analyses were performed on the FACScan flow cytometer.

### Real-time PCR

Total RNA was isolated using the RNeasy Mini Kit (Qiagen, Germany) according to the manufacturer’s instructions. For mRNA detection, reverse transcription of RNA was performed using the ReverTra Ace qPCR RT Kit (TOYOBO, Osaka, Japan), and real-time PCR (RT-PCR) was carried out using SYBR® Premix Ex Taq™ II (Tli RNaseH Plus) (Takara, Kusatsu, Shiga, Japan). The primers are listed in Supplementary Table. The process was detailed in our previous study^40^.

### Western blotting

The cellular lysate was fractionated by 12% SDS-PAGE and electroblotted onto PVDF membranes. Membranes were incubated with primary antibodies including anti-Dicer1 (rabbit, ABclonal, USA), anti-p21 (rabbit, ABclonal, USA) and anti-βactin (rabbit, ABclonal, USA). Secondary goat anti-rabbit antibodies labeled with horseradish peroxidase (Amersham Biosciences) were used. The process was detailed in our previous study^17, 41^.

### Dicer1 shRNA transductions

The HC-MSCs were transfected with shDicer1-eGFP vectors (shRNA group) and negative vector. And detailed information about cell transductions is provided in our previous articles^17^.

### Cell transductions of adenoviral vectors for expression of Dicer1

The process of the construction of adenoviral vectors for expression of Dicer1 was detailed previously^17, 41^. The MM-MSCs were planted in 6-well plates and cultivated for 24 h. The cells were transfected with 20 μl adenoviral vectors and cultured for 12 h and then the medium was changed. The cells were cultured for another 36 h and harvested. The transfection was performed according to the manufacturer's protocol.

### MicroRNA-93/microRNA-20a overexpression

MM-MSC were transfected with human miR-20a, miR-93 and scrambled control lentivirus (Genechem Company, Shanghai, China) according to the manufacturer’s protocol.

### Statistical analysis

The data were presented as mean ± SD. All statistical analyses were performed using the SPSS 17.0 System. Comparison of mRNA levels between healthy controls and different MM subtypes was using a Student’s *t* test, one-way analysis of variance (ANOVA) was used to assess multiple pairwise comparisons *p* < 0.05 was considered statistically significant. Estimation of PFS was performed using the method of Kaplan and Meier.

## Electronic supplementary material

Supplementary Table

Supplementary legend

Figure S1

Figure S2

Figure S3

Figure S4
