# Supplementary material for: Dicer1 downregulation by multiple myeloma cells promotes the senescence and tumor-supporting capacity and decreases the differentiation potential of mesenchymal stem cells
Source: Cell Death Dis. 2018 May 3;9(5):512. doi: 10.1038/s41419-018-0545-6 (PMC5938708; doi:10.1038/s41419-018-0545-6)
Supplement: Supplementary file 1 — Supplementary Table [file 41419_2018_545_MOESM1_ESM.doc]

**Supplementary Table.** The sequence of primers used for real time PCR

| Gene | Forward primer (5' - 3') | Reverse primer (5' - 3') |
| --- | --- | --- |
| GAPDH | GCACCGTCAAGGCTGAGAAC | GTGGTGAAGACGCCAGTGGA |
| RUNX2 | AGTGGACGAGGCAAGAGTTTC | CCTTCTGGGTTCCCGAGGT |
| ALP | CCATTCCCACGTCTTCACATT | AAGGGCTTCTTGTCTGTGTCACT |
| P21 | TGTCCGTCAGAACCCATGC | AAAGTCGAAGTTCCATCGCTC |
| P53 | AGGCCTTGGAACTCAAGGAT | CCCTTTTTGGACTTCAGGTG |
| Dicer1 | TGCTATGTCGCCTTGAATGTT | AATTTCTCGATAGGGGTGGTCTA |
| SDF-1 | ATTCTCAACACTCCAAACTGTGC | ACTTTAGCTTCGGGTCAATGC |
| IL-6 | CCTGAACCTTCCAAAGATGGC | TTCACCAGGCAAGTCTCCTCA |
| DKK-1 | CCTTGAACTCGGTTCTCAATTCC | CAATGGTCTGGTACTTATTCCCG |
| GDF-15 | GACCCTCAGAGTTGCACTCC | GCCTGGTTAGCAGGTCCTC |
