# Supplementary material for: Dicer1 downregulation by multiple myeloma cells promotes the senescence and tumor-supporting capacity and decreases the differentiation potential of mesenchymal stem cells
Source: Cell Death Dis. 2018 May 3;9(5):512. doi: 10.1038/s41419-018-0545-6 (PMC5938708; doi:10.1038/s41419-018-0545-6)
Supplement: Supplementary file 2 — Supplementary legend [file 41419_2018_545_MOESM2_ESM.docx]

**Figure S1.** Progression free survival (PFS) of myeloma patients according to the percentage of SA-β-gal-positive cells detected at diagnosis of MM-MSC. (NS-MM-MSCs, SA-β-gal-positive cells < 4.4%, S-MM-MSCs, SA-β-gal-positive cells ≥ 4.4%).

**Figure S2. Knockdown of Dicer1 inhibited adipogenic differentiation of MSCs.** (A) Representative images of lipid droplet formation after 21 days of adipogenic differentiation (100×magnification). (B, C) Relative FABP4 and C/EBPα mRNA expression levels in cells after differentiation for 7, 14, and 21 days. The results are expressed as means±SD. Compared with controls, the significance was set as **p*≤0.05; ***p*≤0.01.

**Figure S3.** Myeloma cells supporting cytokine expression levels were measured by RT-PCR(A) and ELISA(B) in HC-MSC(n=18) and MM-MSC(n=46). The results are expressed as means ± SD. The average of three replicates is displayed. Compared with controls, the significance was set as * *p*≤0.05; ** *p*≤0.01.

**Figure S4. Upregulation of Dicer1 reversed** **adipogenic differentiation of MM-MSCs.**

(A) Representative images of lipid droplet formation after 21 days of adipogenic differentiation (100×magnification). (B, C) Relative FABP4 and C/EBPα mRNA expression levels in cells after differentiation for 7, 14, and 21 days. The results are expressed as means±SD. Compared with controls, the significance was set as **p*≤0.05; ***p*≤0.01.
