# Supplementary figures and images for: Dicer1 downregulation by multiple myeloma cells promotes the senescence and tumor-supporting capacity and decreases the differentiation potential of mesenchymal stem cells
Source: Cell Death Dis. 2018 May 3;9(5):512. doi: 10.1038/s41419-018-0545-6 (PMC5938708; doi:10.1038/s41419-018-0545-6)

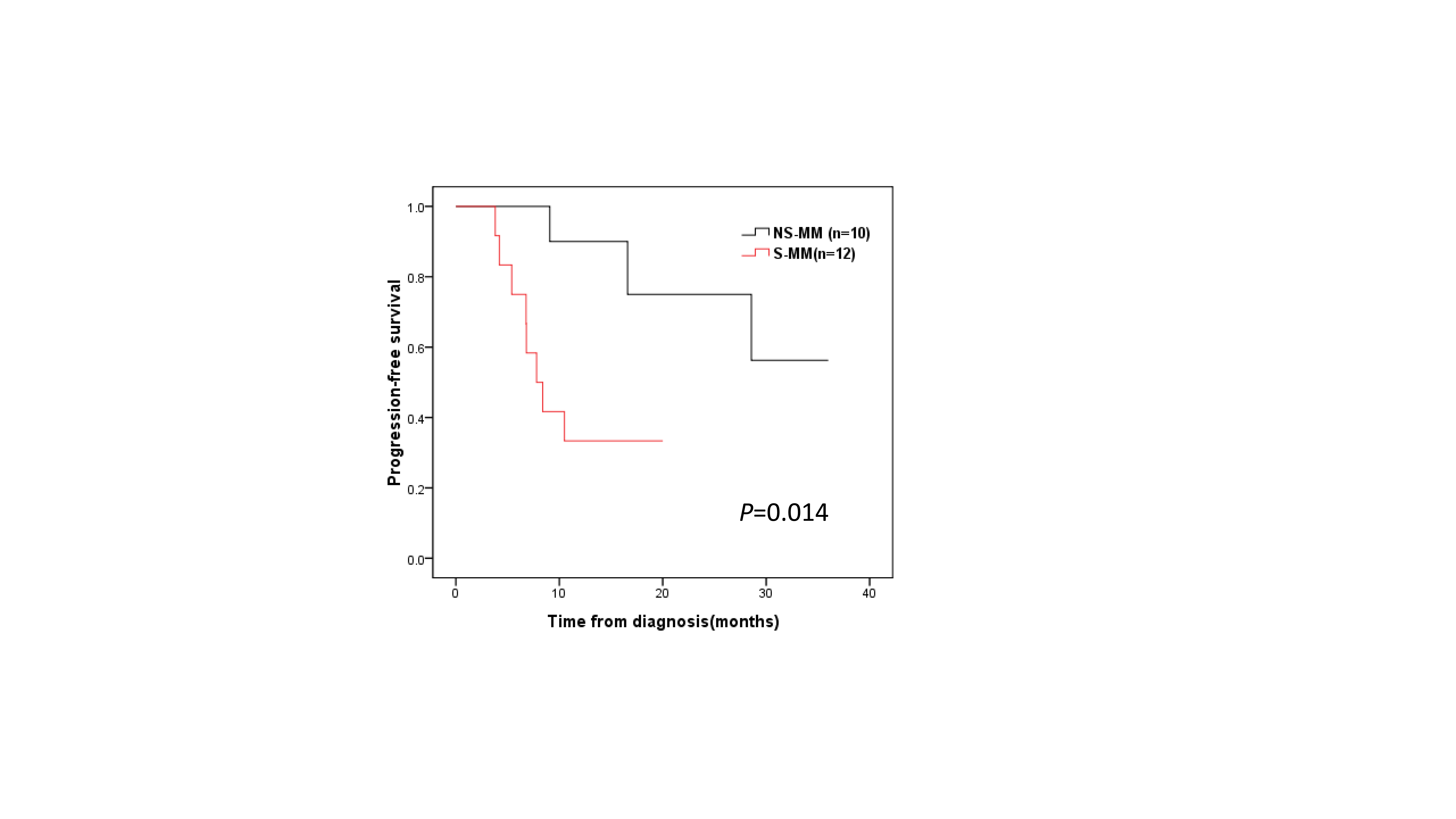

Supplement: Supplementary file 3 — Figure S1 [file 41419_2018_545_MOESM3_ESM.tif]

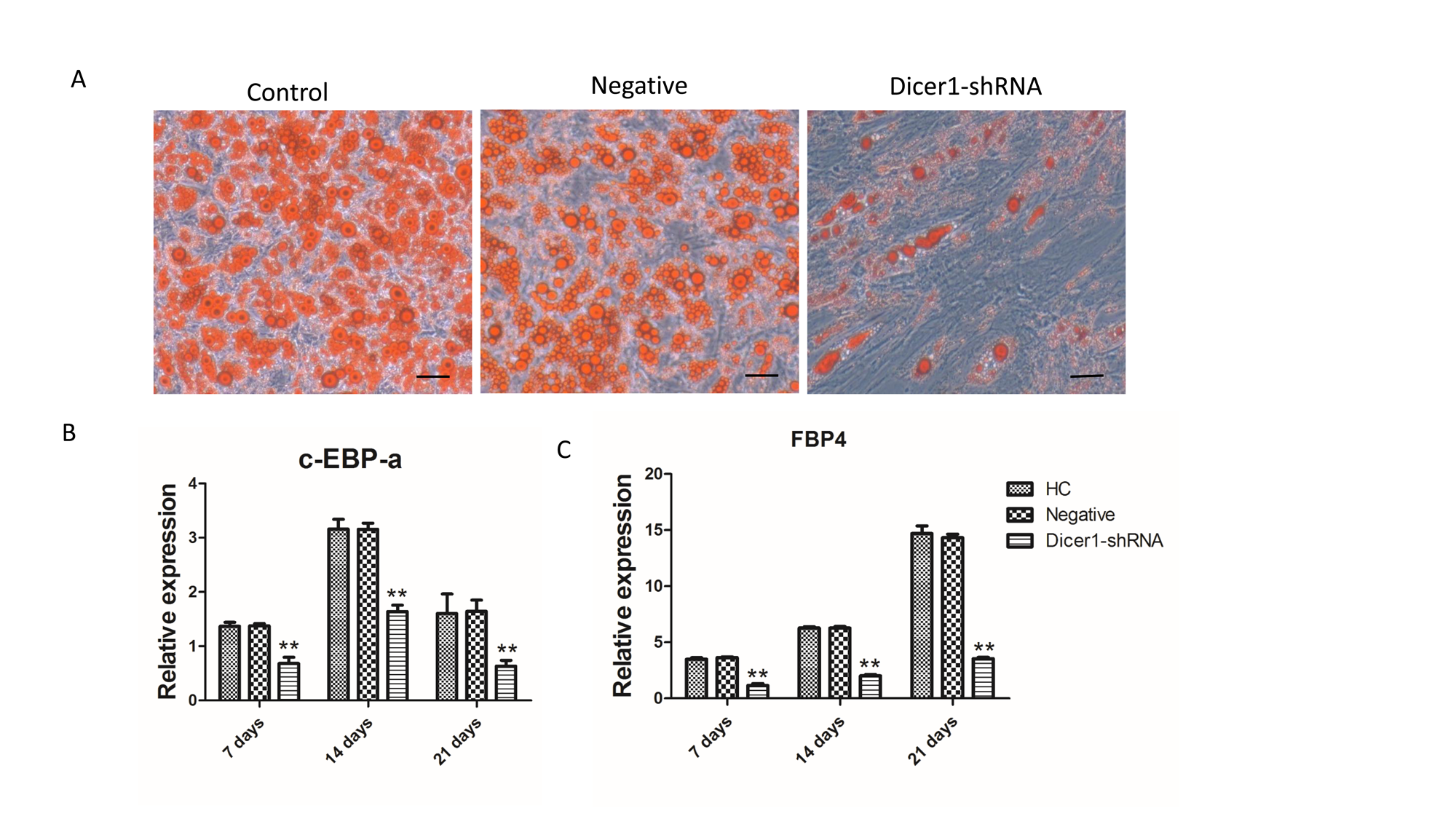

Supplement: Supplementary file 4 — Figure S2 [file 41419_2018_545_MOESM4_ESM.tif]

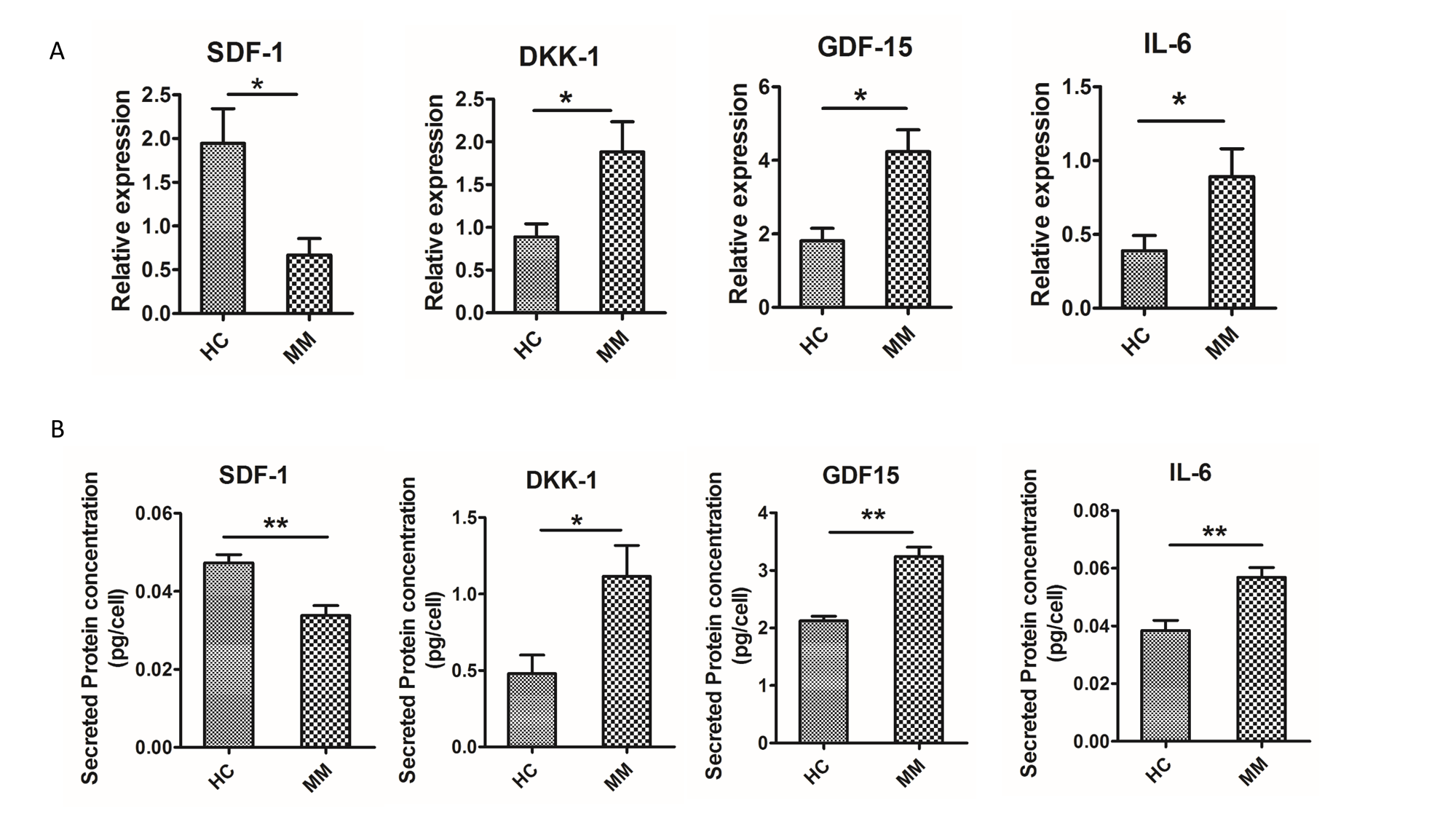

Supplement: Supplementary file 5 — Figure S3 [file 41419_2018_545_MOESM5_ESM.tif]

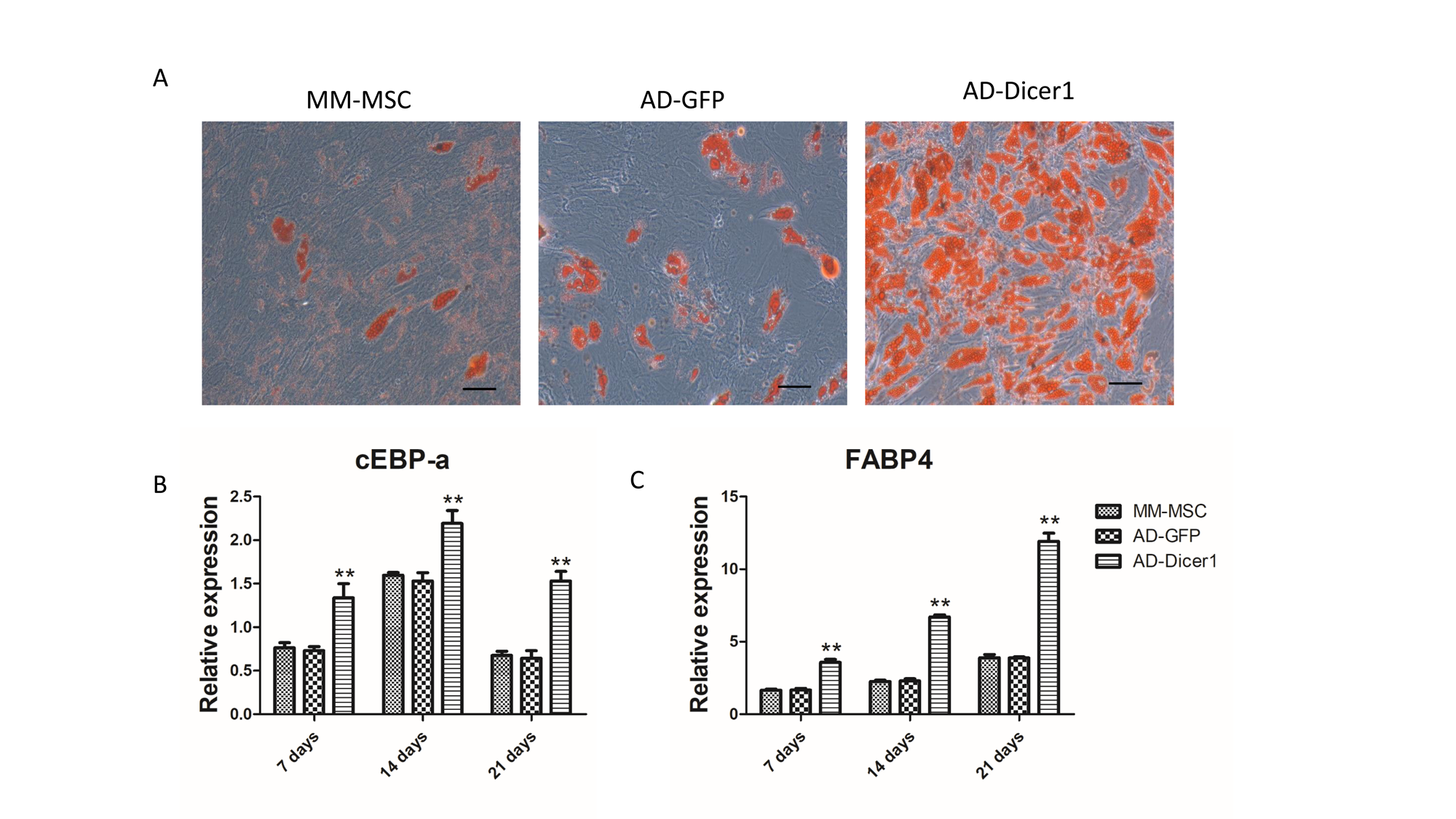

Supplement: Supplementary file 6 — Figure S4 [file 41419_2018_545_MOESM6_ESM.tif]
